# Prediction of sea water intrusion for mining activity in close precincts of sea shore

**DOI:** 10.1186/2193-1801-2-417

**Published:** 2013-08-29

**Authors:** Awanindra Pratap Singh, Prem Kumar Gupta, Manoj Khandelwal

**Affiliations:** Central Institute of Mining and Fuel Research, R. No. 23, Coal Mining & Hydrology Division, Barwa Road, Dhanbad, 826 015 India; Department of Mining Engineering, College of Technology and Engineering, Maharana Pratap University of Agriculture and Technology, Udaipur, 313 001 India

**Keywords:** Sea water intrusion, Ghyben-Herzberg, Surka, Lignite, Electrical resistivity survey

## Abstract

The mining lease area of Surka [District Bhavnagar, Gujarat (India)] is located within 6–12 km horizontal distance of sea shore of Gulf of Cambay. Whenever, there will be onset of lignite extraction, there will be always a threat of sea water intrusion into the mining lease area due to its close proximity to seashore. This article describes the prediction of sea water intrusion into the lease area of whole mining block using Ghyben-Herzberg relation between fresh and saline water, Remote Sensing, Ground Truth verification, Electrical Resistivity Survey and groundwater table monitored during the year 2004. As per the Ghyben-Herzberg relation, results show that there will not be sea water intrusion. If there is excess pumping of water then also the basement rock below the lignite seam will put hindrance to any possible upconing of saline water interface.

## Introduction

**Figure 1:**
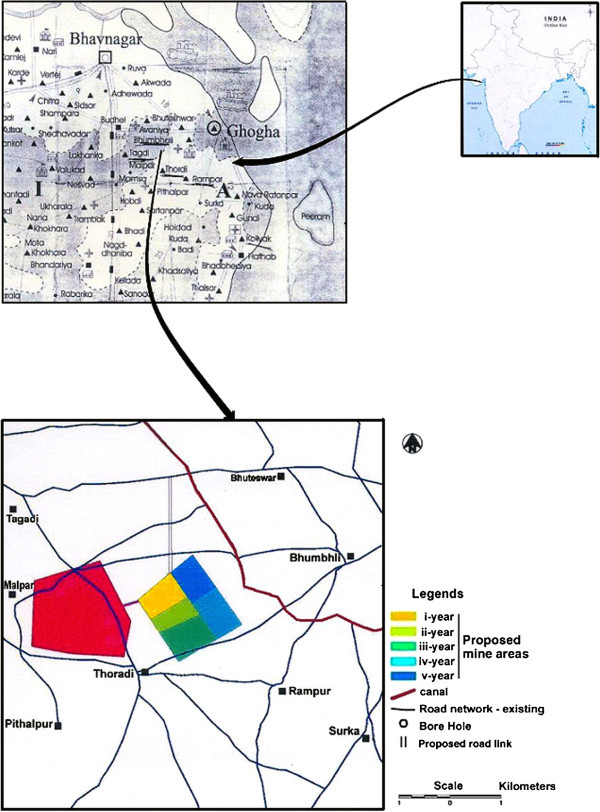
**Location map of the study area.**

**Figure 2:**
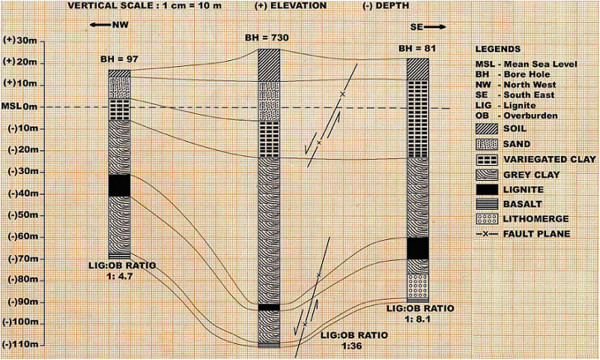
**The generalised cross section over proposed mining block of three drill holes.**

**Figure 3:**
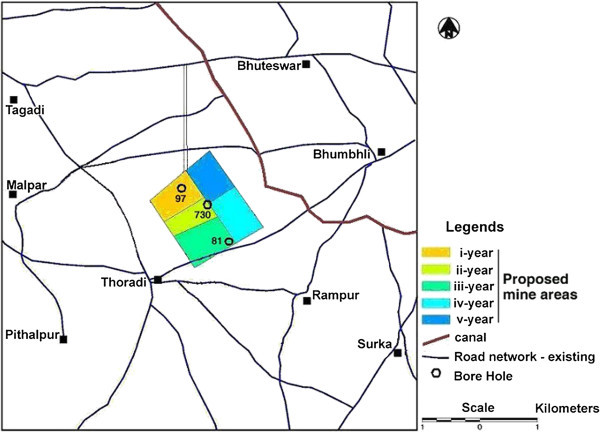
**Locations of drill holes over mining lease area and proposed mining blocks for 1**^**st**^**to 5**^**th**^**year.**

Surka Mining Lease area is located at a distance of 30 km in the south of District Bhavnagar (Gujarat, India) belongs to Gujarat Mineral Development Corporation (GMDC) also shown in the location map of the area as Figure 1. GMDC has plan for Lignite mining at Surka mining lease area. The lignite reserve at Surka has been proved by Commission of Geology and Mining (CGM) by detailed prospecting/exploratory drilling during 1981–2001. GMDC has also conducted exploratory drilling during 2002 with keeping depth range up to 140 m. The generalized cross section over proposed mining block of three drill holes is illustrated in Figure 2 and their locations over mining lease is shown by satellite output as Figure 3. In Surka mining lease area, the disposition of lignite seam ranges between 55–118 m depth from surface and in other words 32–95 m from mean sea level (MSL). Beyond lignite seam, there is presence of grey clay & basalt as the basement rock below lignite seam.

The occurrence of lignite deposit can be categorized into shallow, moderate and higher depth. Shallow depth range is 55–65 m, moderate depth range is 83–93 m and higher depth range is 115–118 m also shown in Figure 2.

Surka mining lease area is having Gulf of Cambay in its eastern side and surrounded by prominent villages namely Thoradi, Tagadi, Rampur, Bhumbhli, Pithalpur, Surka and Bhuteswar, also shown in Figure 3 as a basemap of Surka mining lease area (satellite output). The proposed mining blocks for I^st^ year, II^nd^ year, III^rd^ year, IV^th^ year and V^th^ year are also shown in Figure 3.

GMDC has plan to extract the lignite through opencast mining but also anticipating danger of sea water intrusion into the mining lease area due to lignite mining activity in proposed mining block. To know this, a detailed study has been conducted in which, there was input of ground truth data collection, electrical resistivity survey, conventional method of ground water table monitoring in three seasons of 2004, remote sensing study / utilization of Google Earth software and use of Ghyben-Herzberg relation between sea water and fresh water to predict the sea water intrusion into the mining lease area.

## Methodology

Different methodologies that have been utilized in this are as follows:

Ground Truth Data Collection

In this, the coordinates of different prominent infrastructural setups have been documented with use of Ground Positioning System (GPS).

Electrical Resistivity Survey

Electrical resistivity Survey is an indirect geophysical method. Geophysical methods have been successfully utilized for characterization of physical properties of earth strata where direct method is not feasible (Kate 2007). Resistivity study has been conducted for lithology of Ghogha area (Sharma and Mehta 1979) and their findings are summarized in Table 1.

**Table 1:** **Resistivity value of typical lithology of Ghogha area**

S. no.	Lithology	Resistivity value in Ohm m
1	Sand / gravel	1-5
2	Soil / Stream sediment	8-15
3	Weathered trap (Basalt)	20-60
4	Massive trap (Basalt)	70-250

The Electrical resistivity survey is based on Ohm’s law, which tells the easiness and difficulty of current flow in different or specific media in terms of resistance. The apparent resistivity (Ohm m) is calculated as per following empirical formula (Dobrin 1960):

Where,

ρ_a,_ Apparent resistivity

G, Geometric factor depending upon geo-electrode configuration and geo-mining condition

ΔV, Potential voltage difference

ΔI, Current intensity

### Conventional method of groundwater table monitoring

To monitor groundwater table in unconfined aquifer wells, the simplest conventional method is to lower a rope (tied with 2–4 kg metalled hook at its one end) upto the last extent of well and measure the full length of rope (up to metalled hook) to determine the total depth of well. To know the groundwater column or to know the groundwater table, subtract the well rope length from the full length of rope. Thus, it gives the depth of groundwater table from the surface.

### Remote sensing study / Use of Google earth software

Remote Sensing data used for the study is Resourse sat I (25th March 2004) and the Google Earth Software of 2008 Europa Technologies, 2008 Tele Atlas and 2008 DMapas has been used.

### Ghyben-Herzberg relation between sea water and fresh water

Two investigators working independently along the Europian coast found that salt water occurred underground not at sea level but at a depth below sea level of about 40 times the height of the fresh water above sea level. This distribution was attributed to hydrostatic equilibrium existing between the two fluids of different densities. The equation derived to explain the phenomenon is generally referred to as the Ghyben-Herzberg relation after its originators (Todd 1980) that is1

Where

h_f_ = Fresh groundwater table column above the mean sea level and

**Figure 4:**
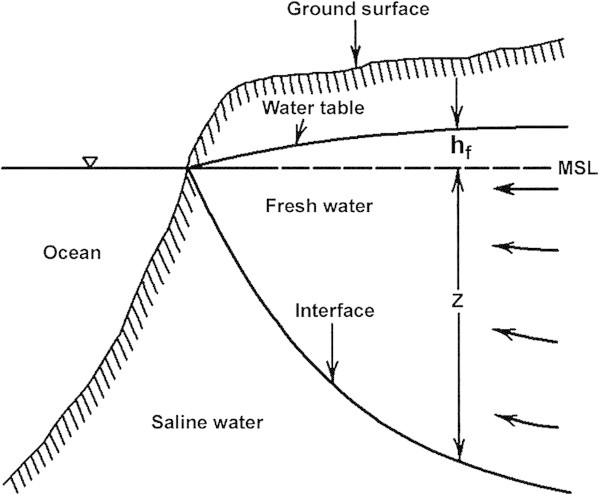
**Idealised sketch of occurrence of fresh and saline groundwater in an unconfined aquifer.**

Z = Depth of fresh water and saline water interface below the mean sea level 40 times the h_f_ also shown in Figure 4 which is the Idealized sketch of occurrence of fresh and saline groundwater in an unconfined aquifer.

## Results and discussions

The satellite data of Resource sat I for the area under study has been digitized with demarcation of whole mining block for the I^st^ - V^th^ year mining plan and the dump site as also shown in Figure 1. The mining block is surrounded by some prominent villages namely Bhuteswar, Bhumbhli, Rampur, Thoradi, Surka, Pithalpar and Tagadi. The coordinates of the different villages and the four corners of whole mining block have been obtained through GPS in field as follows:

- Northern end of whole mining block = 21° 40’ 44.77” N, 72° 11’ 56.27” E, Minimum distance from sea shore is 7.9 km
- Western end of whole mining block = 21° 40’ 18.86” N, 72° 11’ 22.90” E, Minimum distance from sea shore is 9.00 km
- Southern end of whole mining block = 21° 39’ 44.08” N, 72° 11’ 48.01” E, Minimum distance from sea shore is 8.86 km
- Eastern end of whole mining block = 21° 40’ 04.02” N, 72° 12’ 26.42” E, Minimum distance from sea shore is 7.6 km
- Bhuteswar = 21° 41’ 29.24” N, 72° 13’ 00.87” E, Minimum distance from sea shore is 5.50 km
- Bhumbhli = 21° 40’ 51.28” N, 72° 13’ 38.07” E, Minimum distance from sea shore is 5.14 km
- Tagadi = 21° 41’ 12.44” N, 72° 10’ 04.23” E, Minimum distance from sea shore is 10.20 km
- Thoradi = 21° 39’ 28.79” N, 72° 11’ 23.48” E, Minimum distance from sea shore is 9.79 km
- Pithalpur = 21° 38’ 45.49” N, 72° 10’ 19.85” E, Minimum distance from sea shore is 11.77 km
- Rampur = 21° 39’ 07.35” N, 72° 12’ 50.78” E, Minimum distance from sea shore is 7.42 km
- Surka = 21° 38’ 47.02” N, 72° 13’ 48.54” E, Minimum distance from sea shore is 6.01 km

The distances are also calculated from the sea shore with the help of Google Earth.

**Figure 5:**
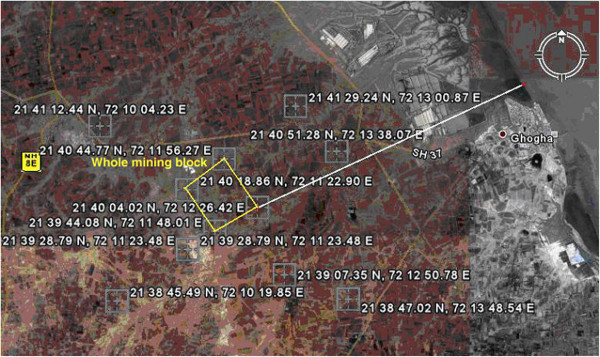
**Coordinates and their respective distances from the sea shore about the whole mining block with respect to the sea shore (Gulf of Cambay).**

All these coordinates are used over Google Earth software and their respective distances from the sea shore have been determined to have an idea about the whole mining block with respect to the sea shore (Gulf of Cambay) as also shown in Figure 5 (produced from Google Earth software).

### Groundwater table (GWT) monitoring

**Figure 6:**
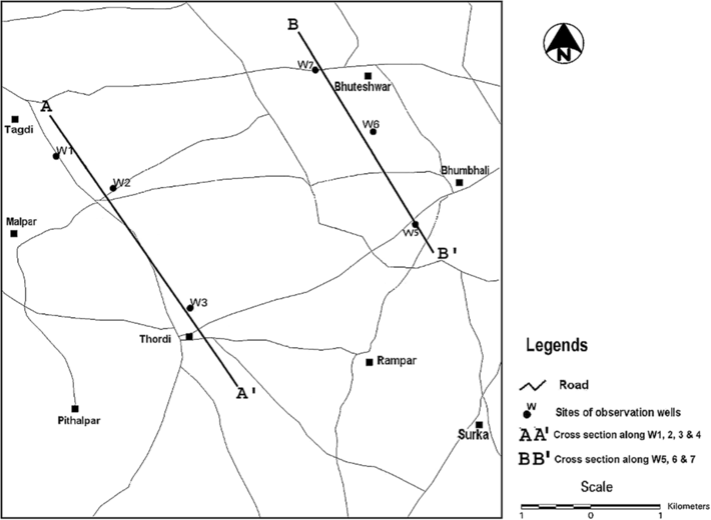
**Locations of W1, W2, W3, W5, W6 and W7 wells.**

Groundwater table has been monitored for three seasons namely pre monsoon, monsoon and post monsoon. Six wells have been selected over the mining lease area, which were also in the surroundings of whole mining block. These wells are W1, W2, W3, W5, W6 and W7. The locations of these wells are shown in Figure 6. The six wells groundwater table monitoring has been tabulated in Table 2.

**Table 2:** **Groundwater table (GWT) fluctuations in three seasons of 2004 of area under study**

S N	Well no.	Reduced level (RL) in m	Pre monsoon		Monsoon		Post monsoon	
			GWT (m)	GWC (m)	GWT (m)	GWC (m)	GWT (m)	GWC (m)
1	W1	31.5	9.9	7.1	3.6	13.4	5	12
2	W2	30	21.2	8.3	18	11.5	17.5	12
3	W3	31	11	7	4.2	13.8	11.6	6.4
4	W5	13.5	3.5	1	1.4	3.1	1.7	2.8
5	W6	14	3.6	5.1	0.5	8.2	1	7.7
6	W7	14	5.4	5.2	1	9.6	4.2	6.4

### Prediction of sea water intrusion

Sea water intrusion prediction has been done for the whole mining block (to be extracted in five years), where lignite seam has been found at the depth range of 55–118 m using Ghyben-Herzberg relation between fresh and saline water.

Groundwater table monitoring data for all the three seasons has been kept as the basic data to evaluate the fresh and saline water interface. For sea water intrusion prediction, following parameters have been calculated:

- Total depth of well (TDW): Calculated by adding groundwater table (GWT) with groundwater column (GWC) in well.
- Total groundwater column above the mean sea level (TGWC or h_f_): Calculated by subtracting groundwater table (GWT) from the reduced level (RL) of the well.

On the basis of above inputs, the fresh and saline water interface below the mean sea level has been calculated using Equation (1) and the results are tabulated in Table 3 for all the three seasons.

**Table 3:** **Calculated fresh and saline water interface for all the three seasons [Here (-) sign before the values of ‘Z’ shows the depth of fresh and saline water interface below the mean sea level]**

**Pre monsoon season**							
**S. No.**	**Well No.**	**RL**	**GWT**	**GWC**	**TDW**	**TGWC(h**_**f**_**)**	**Z=40h**_**f**_
1	W1	31.5	9.9	7.1	17	21.6	-864
2	W2	30	21.2	8.3	29.5	8.8	-352
3	W3	31	11	7	18	20	-800
4	W5	13.5	3.5	1	4.5	10	-400
5	W6	14	3.6	5.1	8.7	10.4	-416
6	W7	14	5.4	5.2	10.6	8.6	-344
**Monsoon season**							
**S. No.**	**Well No.**	**RL**	**GWT**	**GWC**	**TDW**	**TGWC(h**_**f**_**)**	**Z=40h**_**f**_
1	W1	31.5	3.6	13.4	17	27.9	-1116
2	W2	30	18	11.5	29.5	12	-480
3	W3	31	4.2	13.8	18	26.8	-1072
4	W5	13.5	1.4	3.1	4.5	12.1	-484
5	W6	14	0.5	8.2	8.7	13.5	-540
6	W7	14	1	9.6	10.6	13	-520
**Post monsoon season**							
**S. No.**	**Well No.**	**RL**	**GWT**	**GWC**	**TDW**	**TGWC(h**_**f**_**)**	**Z=40h**_**f**_
1	W1	31.5	5	12	17	26.5	-1060
2	W2	30	17.5	12	29.5	12.5	-500
3	W3	31	11.6	6.4	18	19.4	-776
4	W5	13.5	1.7	2.8	4.5	11.8	-472
5	W6	14	1	7.7	8.7	13	-520
6	W7	14	4.2	6.4	10.6	9.8	-392

**Figure 7:**
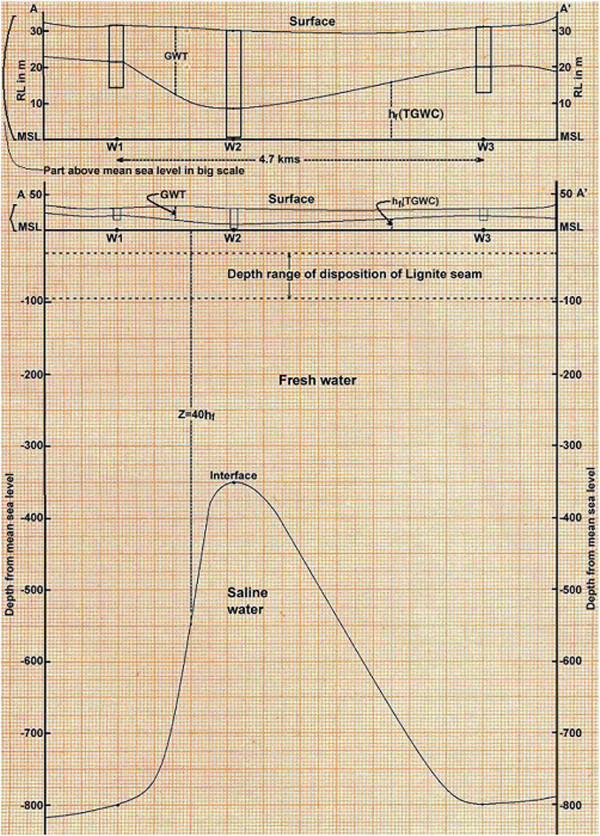
**Cross sections of wells W1, W2 and W3.**

**Figure 8:**
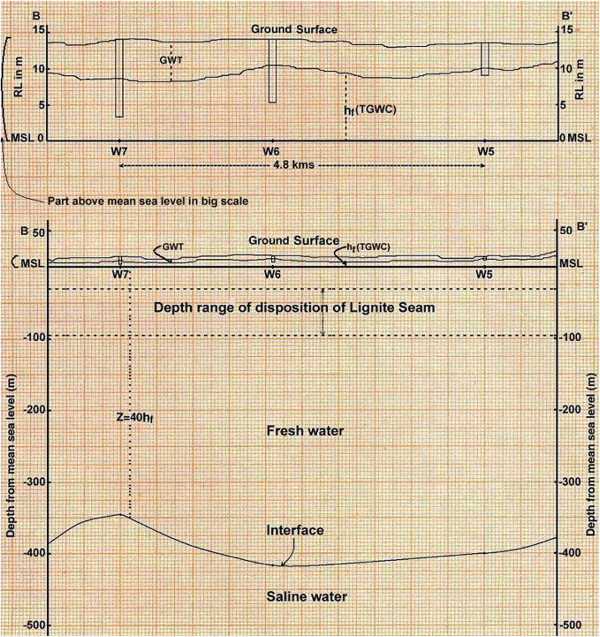
**Cross sections of wells W5, W6 and W7.**

Also, the cross sections have been drawn along the AA’ and BB’ alignments as shown in Figure 6. The AA’ alignment comprises of wells W1, W2 and W3 and its cross section is shown in Figure 7. The BB’ alignment comprises of wells W5, W6 and W7 and its cross section is shown in Figure 8. For cross sections along alignment only pre monsoon season data has been considered, since during pre monsoon season only the ground water level diminishes as much as possible.

As per cross section along alignment AA’ (Figure 7), it is imperative that the maximum depth of lignite seam below the mean sea level is 95 m whereas the minimum depth of fresh and saline water interface against the lignite seam is 352 m below mean sea level. Also in this case, wells W1, W2 and W3 are in western side of whole mining block.

The cross section along alignment BB’ can give much clear picture, since it is in the eastern side of whole mining block. As per the cross section along alignment BB’ (Figure 8), it is imperative that against the maximum depth of lignite seam that is 95 m from mean sea level, the minimum depth of fresh and saline water interface is 344 m from the mean sea level. Therefore, in present scenario there is no such possibility of sea water intrusion. Whenever there will be onset of lignite extraction through opencast mining there may be problem of sea water intrusion due to upconing of saline water interface.

### Upconing in interface

Upconing is a phenomenon which occurs when an unconfined aquifer contains an underlying layer of saline water and is pumped by a well or by bore hole or (in this case) by means of opencast mining the upper fresh water portion of the aquifer, a local rise of the upper layer of the aquifer or saline water interface below the pumping site occurs.

**Figure 9:**
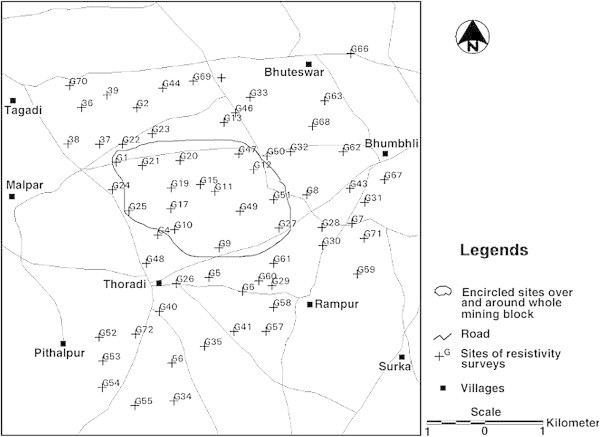
**A view of electrical resistivity survey over the whole mining lease area.**

To know the status of upconing during lignite extraction in whole mining block, electrical resistivity survey has been done over the whole mining lease area as shown in Figure 9.

Resistivity survey has been done specifically to delineate the presence of sub surface impermeable strata beyond the lignite seam. At many sites at different depth ranges clay, sandy clay and massive basalt have been encountered. The depth ranges of above mentioned strata (belongs to only encircled area over and around whole mining block) have been tabulated in following Table 4.

**Table 4:** **Depth ranges of impermeable strata beneath and around whole mining block**

S. N.	Site no.	Depth range (m)	Lithology	Remarks
1	G1	100-140	Basalt	Impermeable
2	G21	170-200	Basalt	Impermeable
3	G24	160-200	Basalt	Impermeable
4	G20	100-130	Basalt	Impermeable
5	G25	130-220	Basalt	Impermeable
6	G19	120-130	Clay	Impermeable
7	G17	180-240	Clay	Impermeable
8	G15	110-200	Clay	Impermeable
9	G47	130-150	Clay	Impermeable
10	G11	80-150	Clay	Impermeable
11	G10	90-140	Sandy horizon	Semi impermeable
12	G12	130-150	Clay formation	Impermeable
13	G49	120-150	Massive basalt	Impermeable
14	G9	150-180	Basalt	Impermeable
15	G51	80-130	Basalt	Impermeable
16	G27	210-240	Sandy clay	Semi impermeable

The depth range of lignite seam is 55–118 m and the depth range of impermeable basalt over and around whole mining block is 80–220 m. Similarly, the depth range of clay formation comes around in the range of 80–240 m and the depth range of sandy clay encountered at two sites in the range of 90–240 m as imperative from the above Table 4.

Therefore, from the whole study it has been established that during the lignite extraction there will not be intrusion of sea water. Even there is not any possibility of upconing of saline water interface. The extraction of lignite would be done only up to the depth range of 55–118 m and the incoming water from the unconfined aquifers would be pumped out of the mine. This may trigger the upconing of fresh and saline water interface but there have been also found impermeable basalt and clay which would prevent such phenomenon to occur.

## Conclusions

From the whole study, following conclusions have been drawn:

- From Remote Sensing, Google Earth software input and ground truth verification data input, it has been found that the minimum distance of actual whole mining block is in the range of 7.59 – 7.85 km (Figure 5), tells the close proximity to seashore.
- As per the Ghyben-Herzberg relation, the saline water interface for the whole mining block has been found at a depth of (−) 344 m against the lignite seam maximum depth of (−) 95 m from the mean sea level in pre monsoon season.
- For the future mining activity, it has been studied and found that there is no possibility of sea water intrusion through upconing of saline water interface due to the presence of impermeable strata in between lignite maximum depth from MSL and undisturbed fresh and saline water interface.
- The findings of this study are very useful for several infrastructural developments and mining activities to know beforehand the status of sea water intrusion.
